# Race-associated biological differences among luminal A and basal-like breast cancers in the Carolina Breast Cancer Study

**DOI:** 10.1186/s13058-017-0914-6

**Published:** 2017-12-11

**Authors:** Humberto Parada, Xuezheng Sun, Jodie M. Fleming, ClarLynda R. Williams-DeVane, Erin L. Kirk, Linnea T. Olsson, Charles M. Perou, Andrew F. Olshan, Melissa A. Troester

**Affiliations:** 1 Division of Epidemiology & Biostatistics, Graduate School of Public Health, San Diego State University, 5500 Campanile Drive, Hardy Tower Room 168, San Diego, CA USA; 2 Department of Epidemiology, University of North Carolina at Chapel Hill, Campus Box 7435, Chapel Hill, NC 27599 USA; 3 Department of Biological and Biomedical Sciences, North Carolina Central University, Durham, NC USA; 4 Department of Genetics, University of North Carolina at Chapel Hill, Chapel Hill, NC USA

**Keywords:** Breast cancer, Gene expression, Disparities, Recurrence

## Abstract

**Background:**

We examined racial differences in the expression of eight genes and their associations with risk of recurrence among 478 white and 495 black women who participated in the Carolina Breast Cancer Study Phase 3.

**Methods:**

Breast tumor samples were analyzed for PAM50 subtype and for eight genes previously found to be differentially expressed by race and associated with breast cancer survival: *ACOX2*, *MUC1*, *FAM177A1*, *GSTT2*, *PSPH*, *PSPHL*, *SQLE*, and *TYMS*. The expression of these genes according to race was assessed using linear regression and each gene was evaluated in association with recurrence using Cox regression.

**Results:**

Compared to white women, black women had lower expression of *MUC1*, a suspected good prognosis gene, and higher expression of *GSTT2*, *PSPHL*, *SQLE*, and *TYMS*, suspected poor prognosis genes, after adjustment for age and PAM50 subtype. High expression (greater than median versus less than or equal to median) of *FAM177A1* and *PSPH* was associated with a 63% increase (hazard ratio (HR) = 1.63, 95% confidence interval (CI) = 1.09–2.46) and 76% increase (HR = 1.76, 95% CI = 1.15–2.68), respectively, in risk of recurrence after adjustment for age, race, PAM50 subtype, and ROR-PT score. Log_2_-transformed *SQLE* expression was associated with a 20% increase (HR = 1.20, 95% CI = 1.03–1.41) in recurrence risk after adjustment. A continuous multi-gene score comprised of eight genes was also associated with increased risk of recurrence among all women (HR = 1.11, 95% CI = 1.04–1.19) and among white (HR = 1.14, 95% CI = 1.03–1.27) and black (HR = 1.11, 95% CI = 1.02–1.20) women.

**Conclusions:**

Racial differences in gene expression may contribute to the survival disparity observed between black and white women diagnosed with breast cancer.

**Electronic supplementary material:**

The online version of this article (doi:10.1186/s13058-017-0914-6) contains supplementary material, which is available to authorized users.

## Background

Historically, white women have had higher incidence rates of breast cancer compared to black women; however, in recent years incidence rates among white and black women have converged [1]. Mortality rates, on the other hand, remain higher among black women, and rates have continued to diverge despite notable improvements in survival in both races since 1990 [2]. Environmental and other factors including socio-economic status, access to and quality of care, and delays in treatment have been cited as potential explanations of the survival disparity, as have biological factors [3]. Previous research indicates that even among estrogen receptor (ER)-positive and HER2-negative breast cancers, which have more favorable outcomes [4], black women have higher mortality rates compared to white women [5]. Recent work highlighted racial differences in risk of recurrence (ROR) scores among ER^+^/HER2^–^ breast cancers [6, 7], but biological differences in tumors between black and white women are only just beginning to be understood.

Several studies have used whole genome expression data to screen for racial differences in tumors [8–11], including our own recent findings [12]. In that study, we examined biological differences by race among luminal A and basal-like breast cancers using publicly available data, and identified several genes including *ACOX2*, *CRYBB2*, *MUC1*, *PSPH*, *SQLE*, and *TYMS* that were differentially expressed by race and that were associated with differences in survival [12]. A limitation of our prior study was the small study population, with data from only 108 Caucasian and 57 African-American women. Herein, we expand this analysis to validate our previous findings in approximately 1000 cases, half of whom are black women, within a larger population-based context. Specifically, we sought to estimate differences in the expression of two suspected good prognosis genes (*ACOX2* and *MUC1*) and six suspected poor prognosis genes (*FAM177A1*, *GSTT2*, *PSPH*, *PSPHL*, *SQLE*, and *TYMS*) by race, and to examine their associations with risk of breast cancer recurrence.

## Methods

### Study population

This study uses data from the Carolina Breast Cancer Study Phase 3 (CBCS3), a population-based study of 3000 women conducted in 24 counties in eastern and central North Carolina from 2008–2013. Recruitment and data collection procedures for CBCS3 and prior study phases appear elsewhere [13]. In brief, women aged 20–74 years residing in the 24 counties and diagnosed with first primary invasive breast cancer were identified using rapid case ascertainment in collaboration with the NC Central Cancer Registry. After determination of study eligibility, sampling was performed to ensure adequate representation of various subgroups (i.e., young and African-American women). After informed consent was obtained, all participants completed an interviewer-administered questionnaire, provided blood samples, and provided written consent for retrieval of medical records and paraffin-embedded tumor blocks.

### Tumor gene expression profiling and molecular subtyping

Procedures for tumor gene expression profiling of the 1013 of 3000 women enrolled in the CBCS3 have been previously published [6]. In brief, RNA was isolated from cores using the Qiagen RNeasy FFPE kit and protocol, with 95% of tumors producing quantifiable RNA. The majority (98.2%) of samples were obtained before neoadjuvant chemotherapy treatment. Samples were randomized to batches for RNA extraction and analyses. In total, 1122 samples from 1042 cases from CBCS3 were analyzed for the PAM50 assay and for the expression of an additional ~150 genes using the NanoString nCounter gene expression system [14]. The PAM50 predictor [15] was used to categorize breast tumors into intrinsic subtype as luminal A, luminal B, HER2-enriched, basal-like, and normal-like, and to calculate the ROR score with proliferation (ROR-P) and tumor size (ROR-PT) included. Probes for nine genes identified by D’Arcy et al. were included: *ACOX2*, *CRYBB2*, *MUC1*, *FAM177A1*, *GSTT2*, *PSPH*, *PSPHL*, *SQLE*, and *TYMS* [12].

Quality control was conducted using the NanoStringNorm package in R. Samples with poor quality were identified using the following criteria: (1) the ratio of the geometric mean expression levels of six positive controls of a sample to the average geometric means of the six positive controls across all samples fell outside the range of 0.3–3; (2) the expression level of 90% of endogenous genes was lower than the mean (+3 SD) of negative controls; and (3) the geometric mean of the reference genes of a sample was greater than 3 SDs from the average geometric means of the reference genes across all batches. Of the 1122 samples, 39 did not pass quality control. We further excluded 70 duplicate samples with lower quality gene expression data, resulting in an analytic gene expression sample of 1013. Of the nine genes of initial interest in the current study, the expression of one (*CRYBB2*) was below the geometric mean of negative controls in > 60% of samples and was not considered further. The raw RNA counts were normalized using the geometric mean of the six positive control genes and then log_2_-transformed for analyses. Among the 1013 women with available gene expression data, we excluded all women who self-identified as non-black or non-Caucasian white, including seven American-Indian, 13 Asian, and 20 women of ‘other’ races, resulting in an analytic sample of 478 white and 495 black women (see Additional file 1: Table S1) for participant characteristics).

### Breast cancer recurrence

The time from breast cancer diagnosis to the first breast cancer recurrence was obtained from the medical records. Among the 973 women with available gene expression data, we identified 114 women with at least one recurrence during a median follow-up of 5.07 years (range = 0.39–8.22 years). Approximately 9% of white women and 15% of black women had at least one recurrence during the follow-up period.

### Statistical analysis

We first examined associations between gene expression and a range of participant demographics, reproductive factors, and clinical characteristics using linear regression and independent sample *t* tests. Based on likelihood ratio tests from age-adjusted linear regression models, with the exception of ER status, there were no significant gene expression-by-covariate interactions. Therefore, results from the independent sample *t* tests based on all women are reported in Additional file 1: Table S1), and age-adjusted RNA counts by race and ER status are reported separately in Additional file 1: Table S2). We then examined race-associated gene expression of the eight genes overall, and by luminal A and basal-like breast cancer subtype using linear regression. In separate models, we regressed the normalized log_2_-transformed expression of each of the eight genes on: race (black vs white), study design variables (age at diagnosis in years, which was used for sampling; and codeset, which varied between Nanostring batches), and PAM50 subtype (luminal A, luminal B, HER2-enriched, basal-like, and normal-like), as appropriate. The covariate-adjusted β coefficients, representing the log_2_(relative difference in gene expression among black women relative to white women), and the corresponding 95% confidence limits from the linear regression models were back-transformed (i.e., 10^(log^_10_^(2)*β)^) to obtain the relative difference in gene expression.

We dichotomized gene expression at the median (i.e., ≤ median = low, and > median = high expression) for each gene and, among the 938 women with breast cancer stages I–III, examined unadjusted associations with risk of recurrence using the Kaplan-Meier survival function. Overall, and by race, and among women with ER^+^/HER2^–^ breast cancer, we used Cox regression to estimate hazard ratios (HRs) and 95% confidence intervals (CIs) for the associations between dichotomized as well as continuous log_2_-transformed gene expression adjusted for age, race, codeset, PAM50 subtype, and ROR-PT score (low, medium, and high), as appropriate. Although breast cancer subtype could potentially mediate the associations between gene expression and breast cancer recurrence, we were interested in understanding these adjusted associations rather than assuming a causal model. We evaluated the joint effects of all eight genes on risk of recurrence by creating a multi-gene race-associated expression (MRE) score. To compute the score, we applied the method of D’Arcy et al. [12] wherein we assigned individual scores of –1 or +1 to each of the eight genes. For six of the eight genes (*FAM177A1*, *GSTT2*, *PSPH*, *PSPHL*, *SQLE*, and *TYMS*), expression below the median was assigned a risk score of –1 indicating lower risk of recurrence, and expression above the median was assigned a score of +1 indicating higher risk of recurrence. Given the inverse associations between survival and expression of *ACOX2* and *MUC1*, for these two genes expression below the median was assigned a score of +1 and expression above the median was assigned a score of –1. We summed the individual gene risk scores resulting in an MRE score ranging from –8 to +8, with higher scores indicating higher risk of recurrence, and also categorized the MRE score as –8 to –2 (low), –1 to 3 (medium), and 4 to 8 (high recurrence risk). We conducted all analyses using SAS version 9.4 (SAS Institute Inc., Cary, NC, USA).

## Results

In this subsample of women from CBCS3, there were approximately equal proportions of black (51%) and white (49%) breast cancer patients (Additional file 1: Table S1). Women were approximately 52 years of age on average, and the majority were postmenopausal (57%), and diagnosed with stage I/II (84%) and grade I/II (52%) tumors. By PAM50 classification, the majority of tumors were luminal A (38%), followed by basal-like (25%), luminal B (20%), HER2-enriched (12%), and normal-like (5%). As previously reported in CBCS3 [6] and elsewhere [5, 7], black women of all ages had a higher frequency of basal-like (33.9% versus 16.7%) and HER2-enriched (13.3% versus 10.0%) cancers, and lower frequency of luminal A breast cancers (29.5% versus 47.3%). Few participant demographic and reproductive factor characteristics were consistently associated with gene expression. On the other hand, the expression of most genes was associated with clinicopathological factors including tumor grade, tumor size, ER and progesterone receptor (PR) status, and PAM50 subtype (Additional file 1: Tables S1 and S2).

### Racial differences in gene expression

Overall, black women had lower expression of *MUC1*, a good prognosis gene, and higher expression *GSTT2*, *PSPHL*, *SQLE*, and *TYMS*, poor prognosis genes, after adjustment for age, codeset, and PAM50 subtype (Table 1). The largest difference in expression was for *PSPHL*, of which black women had expression levels that were more than double those in white women (relative expression = 2.38, 95% CI = 2.11–2.67). Racial patterns in expression of these five genes were similar in direction and magnitude when restricted to women with luminal A breast tumors; however, among women with basal-like tumors, only *GSTT2* and *PSPHL* were differentially expressed by race.

**Table 1:** Racial differences in gene expression among white (*n* = 478) and black (*n* = 495) women, overall and by breast cancer subtype, from CBCS Phase 3, 2008–2013

	Overall (*n* = 973)		Luminal A (*n* = 372)		Basal-like (*n* = 248)	
Gene	Relative expression (95% CI)^a^	*P*	Relative expression (95% CI)^a^	*P*	Relative expression (95% CI)^a^	*P*
*ACOX2*	0.99 (0.87–1.12)	0.849	1.00 (0.86–0.96)	0.958	1.12 (0.79–1.58)	0.539
*MUC1*	0.72 (0.62–0.83)	<0.001	0.68 (0.56–0.82)	<0.001	0.81 (0.58–1.12)	0.202
*FAM177A1*	1.00 (0.94–1.06)	0.941	1.00 (0.94–1.07)	0.962	0.98 (0.80–1.20)	0.834
*GSTT2*	1.41 (1.19–1.68)	<0.001	1.43 (1.10–1.85)	0.008	1.58 (1.07–2.32)	0.020
*PSPH*	1.00 (0.91–1.10)	0.984	0.98 (0.88–1.10)	0.780	1.13 (0.87–1.46)	0.349
*PSPHL*	2.38 (2.11–2.67)	<0.001	2.33 (1.95–2.79)	<0.001	1.77 (1.39–2.24)	<0.001
*SQLE*	1.15 (1.04–1.27)	0.007	1.19 (1.05–1.35)	0.006	0.98 (0.76–1.27)	0.868
*TYMS*	1.20 (1.11–1.29)	<0.001	1.24 (1.12–1.38)	<0.001	1.08 (0.92–1.26)	0.345

RNA counts were normalized log_2_-transformed prior to analysis

*CI* confidence interval

^a^Relative expression comparing black versus white (referent) women; relative expression is adjusted for age, codeset, and PAM50 subtype, as appropriate

### Gene expression and risk of recurrence

**Fig. 1:**
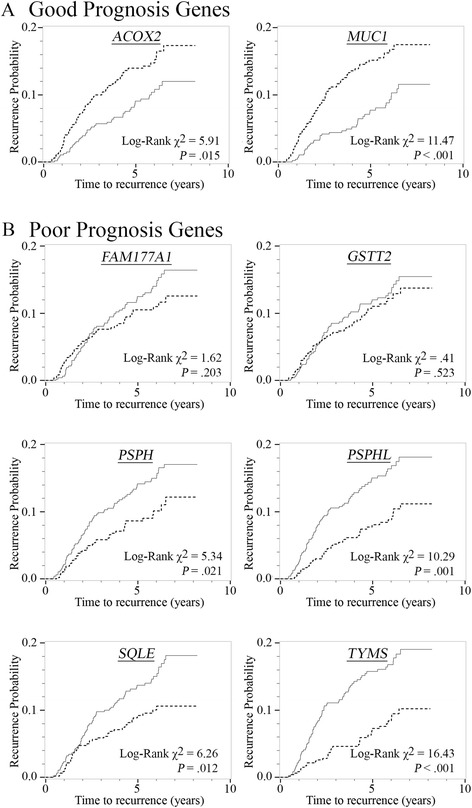
Risk of recurrence by dichotomized gene expression (*dashed line* = expression below median; s*olid line* = expression above median) from CBCS Phase 3, 2008–2013 (*n* = 938). **a** Genes previously found to be inversely associated with breast cancer mortality, and **b** genes previously found to be positively associated with breast cancer mortality

As shown in Fig. 1, among stage I–III women of both races, low (vs high) expression of *ACOX2* and *MUC1* was associated with increased risk of recurrence (log-rank χ^2^ *P* = 0.015 and *P* < 0.001, respectively). In contrast, high (vs low) expression of *PSPH* (*P* = 0.021), *PSPHL* (*P* = 0.001), *SQLE* (*P* = 0.012) and *TYMS* (*P* < 0.001) were each associated with increased risk of recurrence. Most associations with recurrence persisted after adjustment for study design variables (Table 2). Only *FAM177A1*, *PSPH*, and *SQLE* remained statistically significant after further adjustment for PAM50 subtype and ROR-PT score. High expression of *FAM177A1* and *PSPH* was associated with 63% (HR = 1.63, 95% CI = 1.09–2.46) and 76% (HR = 1.76, 95% CI = 1.15–2.68) increases, respectively, in risk of recurrence. Dichotomized *SQLE* expression was not associated with risk of recurrence; however, continuous log_2_-transformed *SQLE* expression was associated with a 20% increase (HR = 1.20, 95% CI = 1.03–1.41) in recurrence risk.

**Table 2:** Gene expression and risk of recurrence, overall and by race, from CBCS Phase 3, 2008–2013

	Overall (*n* = 938)			White (*n* = 465)			Black (*n* = 473)		
Gene	Recurrence/*n*	HR^b^ (95% CI)	HR^c^ (95% CI)	Recurrence/*n*	HR^b^ (95% CI)	HR^c^ (95% CI)	Recurrence/*n*	HR^b^ (95% CI)	HR^c^ (95% CI)
*ACOX2*									
≤ Median	66/469	1.00	1.00	25/206	1.00	1.00	41/263	1.00	1.00
> Median	45/469	0.65 (0.44–0.96)	0.88 (0.58–1.32)	17/259	0.44 (0.24–0.83)	0.68 (0.35–1.32)	28/210	0.84 (0.51–1.37)	1.09 (0.65–1.82)
Log_2_		0.94 (0.83–1.06)	1.05 (0.93–1.18)		0.81 (0.64–1.01)	0.99 (0.79–1.23)		1.01 (0.88–1.17)	1.09 (0.95–1.25)
*MUC1*									
≤ Median	72/474	1.00	1.00	24/179	1.00	1.00	48/295	1.00	1.00
> Median	39/464	0.57 (0.38–0.85)	0.89 (0.57–1.40)	18/286	0.38 (0.21–0.71)	0.62 (0.30–1.26)	21/178	0.71 (0.43–1.19)	1.04 (0.58–1.86)
Log_2_		0.87 (0.79–0.95)	0.97 (0.87–1.08)		0.77 (0.67–0.88)	0.88 (0.74–1.04)		0.93 (0.83–1.05)	1.02 (0.89–1.18)
*FAM177A1*									
≤ Median	47/468	1.00	1.00	16/211	1.00	1.00	31/257	1.00	1.00
> Median	64/470	1.32 (0.89–1.95)	1.63 (1.09–2.46)	26/254	1.05 (0.53–2.06)	1.37 (0.69–2.73)	38/216	1.44 (0.89–2.35)	1.73 (1.04–2.87)
Log_2_		1.14 (0.86–1.50)	1.33 (1.01–1.73)		1.14 (0.68–1.92)	1.46 (0.90–2.39)		1.14 (0.81–1.59)	1.28 (0.92–1.77)
*GSTT2*									
≤ Median	51/471	1.00	1.00	22/250	1.00	1.00	29/221	1.00	1.00
> Median	60/467	1.06 (0.72–1.55)	1.27 (0.86–1.88)	20/215	0.97 (0.53–1.78)	1.26 (0.68–2.34)	40/252	1.18 (0.72–1.93)	1.36 (0.82–2.26)
Log_2_		1.01 (0.92–1.11)	1.07 (0.97–1.18)		0.96 (0.82–1.12)	1.03 (0.88–1.20)		1.06 (0.93–1.20)	1.11 (0.97–1.26)
*PSPH*									
≤ Median	41/472	1.00	1.00	13/228	1.00	1.00	28/244	1.00	1.00
> Median	70/466	1.71 (1.12–2.61)	1.76 (1.15–2.68)	29/237	1.79 (0.87–3.71)	2.04 (1.00–4.15)	41/229	1.66 (0.98–2.80)	1.69 (1.00–2.85)
Log_2_		1.18 (1.00–1.41)	1.18 (1.00–1.39)		1.32 (0.95–1.84)	1.36 (0.98–1.87)		1.15 (0.94–1.40)	1.14 (0.94–1.38)
*PSPHL*									
≤ Median	40/474	1.00	1.00	25/341	1.00	1.00	15/133	1.00	1.00
> Median	71/464	1.52 (0.95–2.44)	1.33 (0.83–2.15)	17/124	1.74 (0.93–3.26)	1.53 (0.80–2.92)	54/340	1.42 (0.72–2.81)	1.24 (0.62–2.48)
Log_2_		1.14 (1.00–1.31)	1.09 (0.95–1.26)		1.19 (0.96–1.48)	1.10 (0.87–1.39)		1.16 (0.97–1.38)	1.10 (0.92–1.32)
*SQLE*									
≤ Median	41/468	1.00	1.00	17/273	1.00	1.00	24/195	1.00	1.00
> Median	70/470	1.47 (0.99–2.17)	1.09 (0.73–1.64)	25/192	1.97 (1.06–3.65)	1.40 (0.72–2.72)	45/278	1.23 (0.75–2.03)	0.96 (0.58–1.60)
Log_2_		1.31 (1.13–1.52)	1.20 (1.03–1.41)		1.40 (1.09–1.80)	1.21 (0.91–1.59)		1.27 (1.06–1.53)	1.19 (0.98–1.44)
*TYMS*									
≤ Median	35/469	1.00	1.00	19/282	1.00	1.00	16/187	1.00	1.00
> Median	76/469	1.93 (1.27–2.92)	1.11 (0.67–1.84)	23/183	1.70 (0.91–3.18)	0.65 (0.29–1.47)	53/286	2.09 (1.18–3.68)	1.50 (0.77–2.90)
Log_2_		1.37 (1.15–1.64)	1.04 (0.83–1.30)		1.38 (1.02–1.85)	0.84 (0.61–1.15)		1.34 (1.06–1.69)	1.08 (0.80–1.46)
MRE Score^a^									
–8 to –2	25/367	1.00	1.00	9/207	1.00	1.00	16/160	1.00	1.00
–1 to 3	36/336	1.48 (0.88–2.48)	1.14 (0.67–1.94)	16/169	1.93 (0.83–4.47)	1.67 (0.72–3.85)	20/167	1.18 (0.61–2.30)	0.88 (0.44–1.76)
4 to 8	50/235	3.03 (1.80–5.07)	2.10 (1.22–3.62)	17/89	4.42 (1.89–10.32)	2.88 (1.17–7.11)	33/146	2.44 (1.29–4.62)	1.78 (0.91–3.49)
Trend		1.15 (1.09–1.22)	1.11 (1.04–1.19)		1.19 (1.08–1.31)	1.14 (1.03–1.27)		1.14 (1.05–1.23)	1.11 (1.02–1.20)

RNA counts were normalized log_2_-transformed prior to analysis; analyses exclude women with unknown stage and stage IV breast cancer

Log2 = continuous normalized log2-transformed gene expression

*CI* confidence interval, *HR* hazard ratio

^a^Multi-gene race-associated expression (MRE) score based on eight genes with higher scores indicating worse risk or recurrence: for *ACOX2* and *MUC1*, ≤ median = 1 vs > median = –1; for *FAM177A1*, *GSTT2*, *PSPH*, *PSPHL*, *SQLE*, and *TYMS*, ≤ median = –1 vs > median = 1

^b^Adjusted for age, race (black vs. white), and codeset, as appropriate

^c^Adjusted for age, race (black vs. white), codeset, PAM50 subtype (luminal A, luminal B, HER2-enriched, basal-like, or normal-like), and risk of recurrence score with tumor size (ROR-PT; low, medium, or high), as appropriate

We next stratified these survival relationships by race. Patterns of recurrence were similar when adjusting for study design factors only. However, after further adjustment for PAM50 subtype and ROR-PT score, most associations among white women were weaker than those among black women, with the exception of *PSPH* which was stronger in white (HR = 2.04, 95% CI = 1.00–4.15) than black (HR = 1.69, 95% CI = 1.00–2.85) women. Among black women, high (vs low) expression of *FAM177A1* was associated with a 73% increase (HR = 1.73, 95% CI = 1.04–2.87) in risk of recurrence in the fully adjusted model.

Breast cancer mortality disparities are greatest among women diagnosed with ER^+^/HER^–^ breast cancer stages I–III; therefore, we assessed survival associations among patients with this clinical subtype. In association with log_2_-unit increase in expression of *MUC1*, white women had reduced risk of recurrence (HR = 0.81, 95% CI = 0.65–1.01), but *MUC1* levels were not associated with recurrence in black women (HR = 0.98, 95% CI = 0.81–1.20). High (vs low) expression of *PSPH* and *TYMS* was associated with more than twice the risk of recurrence in black (*PSPH* HR = 2.25, 95% CI = 0.99–5.13; *TYMS* HR = 2.64, 95% CI = 1.00–6.95), but not white (*PSPH* HR = 1.90, 95% CI = 0.73–4.95; *TYMS* HR = 0.51, 95% CI = 0.19–1.43) women (Additional file 1: Table S3).

The MRE score, which evaluated the additive effects of all eight genes, was associated with increased risk of recurrence among all women (HR = 1.11, 95% CI = 1.04–1.19) and among white (HR = 1.14, 95% CI = 1.03–1.27) and black (HR = 1.11, 95% CI = 1.02–1.20) women after covariate adjustment, including adjustment for PAM50 subtype and ROR-PT score. Among women of both races, the risk of recurrence for women with the highest MRE scores (4 to 8), relative to those with the lowest scores (–8 to –2), was associated with a 110% increase (HR = 2.10, 95% CI = 1.22–3.62) in recurrence risk. In the subgroup of women with ER^+^/HER^–^ breast cancer, the MRE score was associated with a 15% increase in risk in black women (HR = 1.15, 95% CI = 1.00–1.31), but not white women (HR = 1.08, 95% CI = 0.95–1.23).

## Discussion

Previously reported race and survival-associated genes including *MUC1*, *GSTT2*, *PSPHL*, *SQLE*, and *TYMS* were associated with race in this population-based study of women diagnosed with breast cancer. Except for *FAM177A1* and *GSTT2*, the genes we examined in this study were associated with risk of recurrence in unadjusted models. Of the genes differentially expressed by race, *SQLE* expression as a continuous measure was associated with increased risk of breast cancer recurrence, even after adjustment for breast cancer subtype and ROR score. Additionally, a multi-gene score comprised of all eight genes examined in this study was strongly associated with recurrence risk among all women and among black women diagnosed with ER^+^/HER^–^ breast cancer.

Our findings are consistent with prior studies reporting lower expression of *MUC1* and higher expression of *GSTT2*, *PSPHL*, *SQLE*, and *TYMS* among black women compared to white women [9–12]. *MUC1* expression was positively associated with lower grade, smaller tumor size, and positive ER/PR status in our study and in a previous study [16]; however, expression was not associated with recurrence among black women after adjustment for PAM50 subtype, although there was a suggestive inverse association with recurrence among white women. *MUC1*, which is part of a large family of mucin glycoproteins, is involved with cell signaling and cell-cell and cell-matrix adhesion [17], and may impact breast cancer recurrence via these pathways or by directly binding to and activating ERα [18]. In contrast to previous studies, in our study *PSPH* and *ACOX2* were not differentially expressed by race, although *PSPH*, but not *ACOX2*, expression was associated with recurrence. Interestingly, recent evidence suggests that racial differences in the expression of *PSPHL* may be a consequence of a 30-kb deletion from chromosome 7p11, including the promoter and first three of four exons of PSPHL, effectively eliminating *PSPHL* expression, more frequently found among individuals of African ancestry [19]. Although we did not examine *PSPHL* polymorphisms, our findings may reflect underlying genetic differences. Whereas the study by Rummel and colleagues [19] found no association between *PSPHL* loss or retention and pathological characteristics, in our study, *PSPHL* expression was associated with grade, tumor size, ER/PR status, and breast cancer PAM50 subtype [19].

*SQLE* expression was higher in tumors of black women compared to white women, and was associated with more aggressive tumors including tumors of high histologic grade, nodal involvement, larger size, ER^–^/HER2^+^ status, and with increased risk of breast cancer recurrence, consistent with prior studies [20]. Applying the criteria proposed by D’Arcy et al. [12] for a disparity-associated gene that: (1) the gene should be differentially expressed by race in the tumor, and (2) the differential expression of a candidate gene should be associated with a difference in breast cancer survival, we identified *SQLE* as a disparity-associated gene. *SQLE* is located on chromosome 8q24.13, and encodes squalene epoxidase, an enzyme that catalyzes the first oxygenation step in cholesterol synthesis [21]. Given that squalene epoxidase is thought to be one of the rate-limiting enzymes in the cholesterol synthesis pathway, overexpression of *SQLE* may also result in increased cholesterol bioavailability, which may promote ER-dependent growth and Liver X receptor-dependent metastasis [22]. Furthermore, as prior researchers have hypothesized [20], *SQLE* expression together with overexpression of other nearby genes including *RAD21*, which encodes a protein involved in DNA repair, could work to promote a more aggressive cancer phenotype. If *SQLE* is confirmed by other studies, these findings provide further evidence for the potential use of statins in adjuvant breast cancer therapy [23, 24] as well as the potential for *SQLE* inhibition as a novel cancer treatment option [20]. The function of *FAM177A1* (family with sequence similarity 177 member A1) [25] and *PSPHL* (phospherine phosphatase-like) [26] are not well characterized and thus their associations with recurrence are not entirely clear. *PSPHL* is hypothesized to influence rates of cellular proliferation [27], and therefore could potentially directly impact cancer progression.

This study had several strengths including the large population-based design including the oversampling of young and black women; however, this study had several limitations. First, in our analyses of breast cancer recurrence, the proportion of women with at least one recurrence was relatively small (10%); however, ours is the largest study conducted to date on the topic and provides results consistent with previous studies. Second, a limitation of this research is that we cannot establish the mechanism for higher expression levels (i.e., we cannot distinguish between expression changes that are due to differentiation state or cell lineage versus those that are due to tumor-specific mutations). We also note that some of the genes had prognostic value only within one subtype. For example, genes that tend to be strongly associated with proliferation, such as *MUC1* and *TYMS*, tended to have more prognostic value among luminal breast cancers where proliferation status is variable; very few basal-like breast cancers have low proliferation and therefore proliferation genes often do not provide prognostic value. Third, given prior reports of higher expression of *CRYBB2* among black women compared with white women [7–9, 11, 12], we were a priori interested in including *CRYBB2* in our analyses; unfortunately, we were unable to examine expression of this gene due to a large amount of missing data. Future studies should continue to examine *CRYBB2* expression for its potential relevance as a disparity-associated gene. Finally, in this study we did not compare gene expression in tumor tissue to normal or adjacent-normal tissue; however, in our previous work [12] we observed that patterns of expression, comparing normal to tumor, were similar between black and white women. This suggests that differences in cellular composition between black and white women are not responsible for the racial differences in *MUC1* expression.

## Conclusions

In summary, we validated previously observed racial differences in the expression of several genes using a large population-based study. Of the genes that were differentially expressed by race, high expression of one gene, *SQLE*, was also associated with an increased risk of breast cancer recurrence and thus may be a potential disparity-associated gene. Among women with the more favorable ER^+^/HER2^–^ breast cancer subtype, the multi-gene race-associated score comprised of all eight genes was associated with a 15% increase in risk of recurrence among black but not white women. We conclude that racial differences in gene expression may contribute to the survival disparity observed between black and white women diagnosed with breast cancer.

## Additional file

Additional file 1: Table S1. Gene expression by participant and clinical characteristics from CBCS Phase 3, 2008–2013. **Table S2.** RNA counts overall and by race and estrogen receptor (ER) status from CBCS Phase 3, 2008–2013. **Table S3.** Gene expression and risk of recurrence among women with ER^+^/HER2^–^ breast cancer from CBCS Phase 3, 2008–2013. (DOCX 81 kb)

## Abbreviations

*ACOX2*: Acyl-CoA oxidase 2
CBCS3: Carolina Breast Cancer Study Phase 3
CI: Confidence interval
*CRYBB2*: Crystallin beta B2
ER: Estrogen receptor
*FAM177A1*: Family with sequence similarity 177 member A1
*GSTT2*: Glutathione S-transferase theta 2
HR: Hazard ratio
MRE: Multi-gene race-associated expression
*MUC1*: Mucin 1, cell surface associated
PR: Progesterone receptor
*PSPH*: Phosphoserine phosphatase
*PSPHL*: Phosphoserine phosphatase pseudogene 1
ROR: Risk of recurrence
ROR-P: Risk of recurrence score with proliferation
ROR-PT: Risk of recurrence score with tumor size
*SQLE*: Squalene epoxidase
*TYMS*: Thymidylate synthetase
