# Supplementary material for: Race-associated biological differences among luminal A and basal-like breast cancers in the Carolina Breast Cancer Study
Source: Breast Cancer Res. 2017 Dec 11;19:131. doi: 10.1186/s13058-017-0914-6 (PMC5725885; doi:10.1186/s13058-017-0914-6)
Supplement: Additional file 1: Table S1. — Gene expression by participant and clinical characteristics from CBCS Phase 3, 2008–2013. Table S2. RNA counts overall and by race and estrogen receptor (ER) status from CBCS Phase 3, 2008–2013. Table S3. Gene expression and risk of recurrence among women with ER+/HER2– breast cancer from CBCS Phase 3, 2008–2013. (DOCX 81 kb) [file 13058_2017_914_MOESM1_ESM.docx]

**Table S1.** Gene expression by participant and clinical characteristics – CBCS Phase 3, 2008-2013.

|  |  |  | ***ACOX2*** |  | ***MUC1*** |  | ***FAM177A1*** |  | ***GSTT2*** |  | ***PSPH*** |  | ***PSPHL*** |  | ***SQLE*** |  | ***TYMS*** |
| --- | --- | --- | --- | --- | --- | --- | --- | --- | --- | --- | --- | --- | --- | --- | --- | --- | --- |
|  | **n (%)** |  | **Mean (SE)** |  | **Mean (SE)** |  | **Mean (SE)** |  | **Mean (SE)** |  | **Mean (SE)** |  | **Mean (SE)** |  | **Mean (SE)** |  | **Mean (SE)** |
| Race |  |  |  |  |  |  |  |  |  |  |  |  |  |  |  |  |  |
| White | 478 (49.1) |  | 5.23 (.07) |  | 10.21 (.08) |  | 8.92 (.03) |  | 4.99 (.09) |  | 5.75 (.05) |  | 7.87 (.07) |  | 8.64 (.05) |  | 8.41 (.05) |
| Black | 495 (50.9) |  | 4.99 (.08) |  | 9.34 (.09) |  | 8.83 (.04) |  | 5.31 (.09) |  | 5.77 (.06) |  | 9.21 (.09) |  | 8.98 (.06) |  | 8.93 (.05) |
| *P* |  |  | .018 |  | <.001 |  | .075 |  | .012 |  | .854 |  | <.001 |  | <.001 |  | <.001 |
| Age at diagnosis (years) |  |  |  |  |  |  |  |  |  |  |  |  |  |  |  |  |  |
| <50 | 467 (48.0) |  | 4.88 (.07) |  | 9.80 (.09) |  | 8.88 (.03) |  | 5.06 (.09) |  | 5.70 (.06) |  | 8.45 (.09) |  | 8.83 (.05) |  | 8.72 (.05) |
| ≥50 | 506 (52.0) |  | 5.31 (.07) |  | 9.74 (.09) |  | 8.87 (.04) |  | 5.24 (.09) |  | 5.82 (.05) |  | 8.65 (.08) |  | 8.80 (.06) |  | 8.64 (.05) |
| *P* |  |  | <.001 |  | .604 |  | .926 |  | .147 |  | .119 |  | .095 |  | .642 |  | .243 |
| Menopausal status |  |  |  |  |  |  |  |  |  |  |  |  |  |  |  |  |  |
| Premenopausal | 420 (43.2) |  | 4.90 (.08) |  | 9.85 (.09) |  | 8.87 (.03) |  | 5.07 (.10) |  | 5.71 (.06) |  | 8.46 (.10) |  | 8.85 (.06) |  | 8.72 (.06) |
| Postmenopausal | 553 (56.8) |  | 5.26 (.07) |  | 9.70 (.08) |  | 8.88 (.04) |  | 5.21 (.09) |  | 5.80 (.05) |  | 8.62 (.08) |  | 8.79 (.05) |  | 8.64 (.04) |
| *P* |  |  | <.001 |  | .214 |  | .873 |  | .300 |  | .254 |  | .182 |  | .432 |  | .207 |
| BMI at diagnosis (kg/m^2^) |  |  |  |  |  |  |  |  |  |  |  |  |  |  |  |  |  |
| <25.0 | 245 (25.3) |  | 5.12 (.10) |  | 10.03 (.12) |  | 8.87 (.05) |  | 5.10 (.13) |  | 5.77 (.08) |  | 8.25 (.12) |  | 8.80 (.07) |  | 8.52 (.07) |
| 25.0–<30.0 | 287 (29.7) |  | 5.09 (.09) |  | 9.78 (.11) |  | 8.89 (.03) |  | 5.29 (.12) |  | 5.76 (.06) |  | 8.54 (.11) |  | 8.88 (.07) |  | 8.71 (.06) |
| ≥30.0 | 436 (45.0) |  | 5.12 (.08) |  | 9.62 (.10) |  | 8.87 (.04) |  | 5.12 (.10) |  | 5.76 (.06) |  | 8.73 (.09) |  | 8.78 (.06) |  | 8.74 (.05) |
| *P* |  |  | .977 |  | .028 |  | .933 |  | .441 |  | .999 |  | .008 |  | .562 |  | .027 |
| Oral contraceptive use |  |  |  |  |  |  |  |  |  |  |  |  |  |  |  |  |  |
| Never | 220 (22.9) |  | 5.11 (.11) |  | 9.85 (.13) |  | 8.95 (.07) |  | 5.11 (.15) |  | 5.89 (.08) |  | 8.61 (.15) |  | 8.95 (.09) |  | 8.73 (.08) |
| Former | 686 (71.2) |  | 5.14 (.06) |  | 9.76 (.07) |  | 8.85 (.03) |  | 5.15 (.07) |  | 5.72 (.04) |  | 8.56 (.07) |  | 8.78 (.04) |  | 8.65 (.04) |
| Current | 57 (5.9) |  | 4.78 (.06) |  | 9.52 (.23) |  | 8.90 (.08) |  | 5.23 (.27) |  | 5.74 (.13) |  | 8.30 (.26) |  | 8.76 (.15) |  | 8.72 (.14) |
| *P* |  |  | .260 |  | .502 |  | .274 |  | .926 |  | .190 |  | .542 |  | .183 |  | .594 |
| Parity |  |  |  |  |  |  |  |  |  |  |  |  |  |  |  |  |  |
| Nulliparous | 130 (13.4) |  | 5.20 (.14) |  | 9.89 (.16) |  | 9.00 (.06) |  | 5.46 (.18) |  | 5.94 (.11) |  | 8.68 (.19) |  | 9.05 (.11) |  | 8.68 (.12) |
| 1 | 196 (20.1) |  | 4.87 (.12) |  | 9.52 (.15) |  | 8.89 (.06) |  | 5.01 (.14) |  | 5.83 (.08) |  | 8.51 (.13) |  | 8.83 (.08) |  | 8.74 (.07) |
| ≥2 | 647 (66.5) |  | 5.16 (.06) |  | 9.82 (.07) |  | 8.85 (.03) |  | 5.13 (.08) |  | 5.70 (.05) |  | 8.54 (.07) |  | 8.76 (.05) |  | 8.66 (.04) |
| *P* |  |  | .069 |  | .112 |  | .115 |  | .137 |  | .068 |  | .705 |  | .040 |  | .652 |
| HRT use |  |  |  |  |  |  |  |  |  |  |  |  |  |  |  |  |  |
| Never | 740 (76.6) |  | 5.04 (.06) |  | 9.71 (.07) |  | 8.86 (.03) |  | 5.19 (.07) |  | 5.74 (.04) |  | 8.63 (.07) |  | 8.84 (.04) |  | 8.74 (.04) |
| Ever | 226 (23.4) |  | 5.34 (.09) |  | 9.95 (.12) |  | 8.92 (.04) |  | 5.07 (.13) |  | 5.85 (.07) |  | 8.29 (.12) |  | 8.74 (.08) |  | 8.49 (.07) |
| *P* |  |  | .014 |  | .105 |  | .370 |  | .446 |  | .211 |  | .017 |  | .303 |  | .002 |
| Stage |  |  |  |  |  |  |  |  |  |  |  |  |  |  |  |  |  |
| I/II | 807 (83.5) |  | 5.12 (.05) |  | 9.75 (.07) |  | 8.87 (.03) |  | 5.15 (.07) |  | 5.74 (.04) |  | 8.51 (.07) |  | 8.80 (.04) |  | 8.67 (.04) |
| IIII/IV | 160 (16.6) |  | 4.99 (.13) |  | 9.80 (.15) |  | 8.90 (.07) |  | 5.24 (.16) |  | 5.91 (.08) |  | 8.76 (.15) |  | 8.94 (.09) |  | 8.74 (.09) |
| *P* |  |  | .352 |  | .732 |  | .669 |  | .605 |  | .091 |  | .143 |  | .173 |  | .428 |
| Grade |  |  |  |  |  |  |  |  |  |  |  |  |  |  |  |  |  |
| I/II | 486 (51.8) |  | 5.47 (.06) |  | 10.33 (.08) |  | 8.96 (.03) |  | 5.27 (.09) |  | 5.76 (.05) |  | 8.29 (.09) |  | 8.51 (.05) |  | 8.24 (.04) |
| IIII | 453 (48.2) |  | 4.70 (.08) |  | 9.15 (.09) |  | 8.80 (.04) |  | 5.07 (.09) |  | 5.80 (.06) |  | 8.86 (.09) |  | 9.16 (.06) |  | 9.17 (.05) |
| *P* |  |  | <.001 |  | <.001 |  | .002 |  | .137 |  | .583 |  | <.001 |  | <.001 |  | <.001 |
| Tumor size (cm) |  |  |  |  |  |  |  |  |  |  |  |  |  |  |  |  |  |
| ≤2.0 | 481 (50.0) |  | 5.22 (.07) |  | 9.97 (.08) |  | 8.90 (.03) |  | 5.22 (.09) |  | 5.68 (.06) |  | 8.36 (.08) |  | 8.74 (.06) |  | 8.49 (.05) |
| >2.0 | 481 (50.0) |  | 4.97 (.08) |  | 9.54 (.09) |  | 8.87 (.04) |  | 5.10 (.10) |  | 5.86 (.05) |  | 8.74 (.09) |  | 8.89 (.05) |  | 8.87 (.05) |
| *P* |  |  | .016 |  | <.001 |  | .432 |  | .390 |  | .020 |  | .003 |  | .041 |  | <.001 |
| Node status |  |  |  |  |  |  |  |  |  |  |  |  |  |  |  |  |  |
| Negative | 555 (57.2) |  | 5.13 (.07) |  | 9.78 (.08) |  | 8.88 (.03) |  | 5.17 (.08) |  | 5.73 (.05) |  | 8.51 (.08) |  | 8.75 (.05) |  | 8.67 (.04) |
| Positive | 415 (42.8) |  | 5.07 (.08) |  | 9.77 (.09) |  | 8.87 (.04) |  | 5.13 (.10) |  | 5.80 (.06) |  | 8.60 (.10) |  | 8.91 (.06) |  | 8.68 (.05) |
| *P* |  |  | .536 |  | .929 |  | .794 |  | .764 |  | .346 |  | .495 |  | .033 |  | .837 |
| ER status |  |  |  |  |  |  |  |  |  |  |  |  |  |  |  |  |  |
| Negative | 256 (26.6) |  | 4.56 (.11) |  | 8.64 (.11) |  | 8.62 (.05) |  | 4.88 (.13) |  | 5.73 (.08) |  | 8.98 (.12) |  | 9.07 (.07) |  | 9.28 (.06) |
| Positive | 707 (73.4) |  | 5.30 (.06) |  | 10.16 (.07) |  | 8.97 (.03) |  | 5.26 (.07) |  | 5.78 (.04) |  | 8.40 (.07) |  | 8.73 (.04) |  | 8.46 (.04) |
| *P* |  |  | <.001 |  | <.001 |  | <.001 |  | .009 |  | .615 |  | <.001 |  | <.001 |  | <.001 |
| PR status |  |  |  |  |  |  |  |  |  |  |  |  |  |  |  |  |  |
| Negative | 340 (35.3) |  | 4.74 (.10) |  | 8.87 (.10) |  | 8.70 (.05) |  | 4.90 (.11) |  | 5.83 (.07) |  | 8.85 (.10) |  | 9.04 (.07) |  | 9.19 (.06) |
| Positive | 623 (64.7) |  | 5.30 (.06) |  | 10.24 (.07) |  | 8.97 (.02) |  | 5.30 (.08) |  | 5.73 (.05) |  | 8.39 (.08) |  | 8.70 (.05) |  | 8.40 (.04) |
| *P* |  |  | <.001 |  | <.001 |  | <.001 |  | .003 |  | .221 |  | <.001 |  | <.001 |  | <.001 |
| HER2 status |  |  |  |  |  |  |  |  |  |  |  |  |  |  |  |  |  |
| Negative | 815 (84.6) |  | 5.14 (.06) |  | 9.74 (.07) |  | 8.88 (.03) |  | 5.14 (.07) |  | 5.78 (.04) |  | 8.51 (.07) |  | 8.76 (.04) |  | 8.67 (.04) |
| Borderline | 14 (1.5) |  | 4.73 (.56) |  | 9.37 (.62) |  | 9.05 (.19) |  | 5.20 (.60) |  | 6.03 (.34) |  | 8.44 (.50) |  | 8.94 (.29) |  | 8.88 (.20) |
| Positive | 134 (13.9) |  | 4.89 (.13) |  | 9.91 (.15) |  | 8.82 (.05) |  | 5.30 (.16) |  | 5.67 (.10) |  | 8.81 (.16) |  | 9.16 (.10) |  | 8.70 (.08) |
| *P* |  |  | .170 |  | .475 |  | .472 |  | .689 |  | .422 |  | .237 |  | .001 |  | .747 |
| PAM50 subtype |  |  |  |  |  |  |  |  |  |  |  |  |  |  |  |  |  |
| Luminal A | 372 (38.2) |  | 5.57 (.06) |  | 10.77 (.07) |  | 8.99 (.03) |  | 5.43 (.10) |  | 5.78 (.05) |  | 8.10 (.09) |  | 8.37 (.05) |  | 7.98 (.04) |
| Luminal B | 193 (19.8) |  | 4.84 (.09) |  | 9.61 (.15) |  | 9.14 (.04) |  | 5.16 (.13) |  | 5.63 (.07) |  | 8.46 (.12) |  | 9.09 (.08) |  | 9.06 (.06) |
| HER2-enriched | 114 (11.7) |  | 5.01 (.17) |  | 9.94 (.14) |  | 8.83 (.06) |  | 5.44 (.21) |  | 5.91 (.13) |  | 9.04 (.22) |  | 9.33 (.12) |  | 8.51 (.11) |
| Basal-like | 248 (25.5) |  | 4.49 (.12) |  | 8.28 (.11) |  | 8.52 (.07) |  | 4.58 (.14) |  | 5.81 (.09) |  | 9.04 (.12) |  | 9.07 (.09) |  | 9.59 (.05) |
| Normal-like | 46 (4.7) |  | 6.07 (.13) |  | 9.92 (.21) |  | 8.86 (.07) |  | 5.26 (.28) |  | 5.55 (.23) |  | 8.72 (.27) |  | 8.53 (.16) |  | 8.13 (.09) |
| *P* |  |  | <.001 |  | <.001 |  | <.001 |  | <.001 |  | .194 |  | <.001 |  | <.001 |  | <.001 |
| *Note:* RNA counts were normalized log_2_-transformed prior to analysis.  *P* values are for independent samples t-tests or one-way ANOVAs, as appropriate. | | | | | | | | | | | | | | | | | |

**Table S2.** RNA counts overall and by race and estrogen receptor (ER) status – CBCS Phase 3, 2008-2013.

|  | ***ACOX2*** |  | ***MUC1*** |  | ***FAM177A1*** |  | ***GSTT2*** |  | ***PSPH*** |  | ***PSPHL*** |  | ***SQLE*** |  | ***TYMS*** |
| --- | --- | --- | --- | --- | --- | --- | --- | --- | --- | --- | --- | --- | --- | --- | --- |
|  | **Mean (SE)** |  | **Mean (SE)** |  | **Mean (SE)** |  | **Mean (SE)** |  | **Mean (SE)** |  | **Mean (SE)** |  | **Mean (SE)** |  | **Mean (SE)** |
| **All Women** | | | | | | | | | | | | | | | |
| ER status |  |  |  |  |  |  |  |  |  |  |  |  |  |  |  |
| Negative | 23.95 (1.07) |  | 430.63 (1.08) |  | 394.12 (1.03) |  | 28.06 (1.09) |  | 53.09 (1.05) |  | 430.84 (1.08) |  | 519.44 (1.05) |  | 589.89 (1.04) |
| Positive | 39.06 (1.04) |  | 1116.45 (1.05) |  | 501.85 (1.02) |  | 38.89 (1.05) |  | 54.93 (1.03) |  | 352.58 (1.04) |  | 428.34 (1.03) |  | 358.19 (1.03) |
|  |  |  |  |  |  |  |  |  |  |  |  |  |  |  |  |
| **White Women** | | | | | | | | | | | | | | | |
| ER status |  |  |  |  |  |  |  |  |  |  |  |  |  |  |  |
| Negative | 25.52 (1.11) |  | 493.74 (1.14) |  | 424.79 (1.05) |  | 25.56 (1.16) |  | 57.84 (1.08) |  | 330.61 (1.13) |  | 570.43 (1.08) |  | 564.80 (1.08) |
| Positive | 40.21 (1.05) |  | 1410.61 (1.06) |  | 499.31 (1.02) |  | 33.31 (1.07) |  | 53.31 (1.04) |  | 216.62 (1.06) |  | 373.51 (1.04) |  | 305.49 (1.04) |
|  |  |  |  |  |  |  |  |  |  |  |  |  |  |  |  |
| **Black Women** | | | | | | | | | | | | | | | |
| ER status |  |  |  |  |  |  |  |  |  |  |  |  |  |  |  |
| Negative | 22.80 (1.09) |  | 359.89 (1.10) |  | 378.33 (1.05) |  | 31.67 (1.11) |  | 51.22 (1.07) |  | 617.25 (1.11) |  | 522.07 (1.07) |  | 646.99 (1.05) |
| Positive | 38.03 (1.07) |  | 880.76 (1.07) |  | 506.07 (1.03) |  | 45.50 (1.08) |  | 56.86 (1.05) |  | 579.31 (1.08) |  | 495.80 (1.05) |  | 422.03 (1.04) |
|  |  |  |  |  |  |  |  |  |  |  |  |  |  |  |  |
| *P_Interaction_* | 0.002 |  | 0.001 |  | 0.088 |  | 0.002 |  | 0.059 |  | 0.026 |  | 0.004 |  | 0.047 |
| *Note:* RNA counts were normalized log_2_-transformed prior to analysis and back-transformed to obtain normalized RNA counts.  *P_Interaction_* values for race-by-ER status interactions based on likelihood ratio tests from age-adjusted linear regression models. | | | | | | | | | | | | | | | |

## Table S3. Gene expression and risk of recurrence among women with ER+/HER2– breast cancer – CBCS Phase 3, 2008-2013.

|  | **Overall (n = 593)** | | | |  | **White (n = 340)** | | | |  | **Black (n = 253)** | | | |
| --- | --- | --- | --- | --- | --- | --- | --- | --- | --- | --- | --- | --- | --- | --- |
|  | **Recurr./n** | **HR^b^ (95% CI)** |  | **HR^c^ (95% CI)** |  | **Recurr./n** | **HR^b^ (95% CI)** |  | **HR^c^ (95% CI)** |  | **Recurr./n** | **HR^b^ (95% CI)** |  | **HR^c^ (95% CI)** |
| ***ACOX2*** |  |  |  |  |  |  |  |  |  |  |  |  |  |  |
| ≤Median | 28/255 | 1.00 |  | 1.00 |  | 14/133 | 1.00 |  | 1.00 |  | 14/122 | 1.00 |  | 1.00 |
| >Median | 25/338 | .68 (.39-1.18) |  | .81 (.45-1.44) |  | 11/207 | .46 (.21-1.02) |  | .67 (.28-1.60) |  | 14/131 | 1.15 (.53-2.49) |  | 1.42 (.62-3.30) |
| Log_2_ |  | 1.00 (.84-1.20) |  | 1.06 (.89-1.26) |  |  | .74 (.54-1.05) |  | .93 (.66-1.32) |  |  | 1.17 (.96-1.44) |  | 1.20 (.98-1.47) |
| ***MUC1*** |  |  |  |  |  |  |  |  |  |  |  |  |  |  |
| ≤Median | 31/246 | 1.00 |  | 1.00 |  | 13/111 | 1.00 |  | 1.00 |  | 18/135 | 1.00 |  | 1.00 |
| >Median | 22/347 | .50 (.29-.89) |  | .71 (.39-1.31) |  | 12/229 | .35 (.16-.79) |  | .55 (.21-1.44) |  | 10/118 | .59 (.27-1.28) |  | .77 (.34-1.76) |
| Log_2_ |  | .83 (.73-.94) |  | .90 (.78-1.04) |  |  | .72 (.60-.87) |  | .81 (.65-1.01) |  |  | .92 (.77-1.10) |  | .98 (.81-1.19) |
| ***FAM177A1*** |  |  |  |  |  |  |  |  |  |  |  |  |  |  |
| ≤Median | 19/258 | 1.00 |  | 1.00 |  | 9/144 | 1.00 |  | 1.00 |  | 10/114 | 1.00 |  | 1.00 |
| >Median | 34/335 | 1.20 (.67-2.17) |  | 1.34 (.73-2.46) |  | 16/196 | .88 (.36-2.15) |  | 1.09 (.44-2.73) |  | 18/139 | 1.46 (.66-3.22) |  | 1.28 (.55-2.97) |
| Log_2_ |  | 1.06 (.71-1.59) |  | 1.12 (.78-1.59) |  |  | .89 (.47-1.67) |  | 1.13 (.64-2.00) |  |  | 1.26 (.74-2.13) |  | 1.18 (.74-1.88) |
| ***GSTT2*** |  |  |  |  |  |  |  |  |  |  |  |  |  |  |
| ≤Median | 26/299 | 1.00 |  | 1.00 |  | 13/182 | 1.00 |  | 1.00 |  | 13/117 | 1.00 |  | 1.00 |
| >Median | 27/294 | .93 (.53-1.61) |  | 1.11 (.62-1.97) |  | 12/158 | .91 (.41-1.99) |  | 1.10 (.49-2.46) |  | 15/136 | 1.14 (.51-2.49) |  | 1.23 (.50-3.01) |
| Log_2_ |  | .99 (.87-1.14) |  | 1.04 (.90-1.19) |  |  | .91 (.74-1.12) |  | .96 (.78-1.18) |  |  | 1.10 (.91-1.33) |  | 1.11 (.91-1.36) |
| ***PSPH*** |  |  |  |  |  |  |  |  |  |  |  |  |  |  |
| ≤Median | 19/291 | 1.00 |  | 1.00 |  | 8/163 | 1.00 |  | 1.00 |  | 11/128 | 1.00 |  | 1.00 |
| >Median | 34/302 | 1.77 (.96-3.27) |  | 1.80 (.97-3.32) |  | 17/177 | 1.40 (.54-3.61) |  | 1.90 (.73-4.95) |  | 17/125 | 2.10 (.94-4.69) |  | 2.25 (.99-5.13) |
| Log_2_ |  | 1.27 (.99-1.63) |  | 1.23 (.96-1.56) |  |  | 1.32 (.80-2.20) |  | 1.51 (.90-2.53) |  |  | 1.28 (.96-1.72) |  | 1.22 (.92-1.61) |
| ***PSPHL*** |  |  |  |  |  |  |  |  |  |  |  |  |  |  |
| ≤Median | 26/336 | 1.00 |  | 1.00 |  | 18/263 | 1.00 |  | 1.00 |  | 8/73 | 1.00 |  | 1.00 |
| >Median | 27/257 | .89 (.45-1.74) |  | .81 (.41-1.60) |  | 7/77 | 1.22 (.51-2.96) |  | 1.12 (.45-2.79) |  | 20/180 | .81 (.32-2.06) |  | .73 (.28-1.91) |
| Log_2_ |  | .96 (.79-1.17) |  | .91 (.74-1.11) |  |  | 1.04 (.77-1.42) |  | .95 (.69-1.31) |  |  | 1.00 (.75-1.33) |  | .89 (.66-1.20) |
| ***SQLE*** |  |  |  |  |  |  |  |  |  |  |  |  |  |  |
| ≤Median | 23/340 | 1.00 |  | 1.00 |  | 13/227 | 1.00 |  | 1.00 |  | 10/113 | 1.00 |  | 1.00 |
| >Median | 30/253 | 1.45 (.83-2.53) |  | .98 (.54-1.78) |  | 12/113 | 1.61 (.73-3.55) |  | 1.05 (.45-2.48) |  | 18/140 | 1.45 (.67-3.16) |  | .91 (.39-2.13) |
| Log_2_ |  | 1.36 (1.09-1.69) |  | 1.19 (.94-1.52) |  |  | 1.32 (.92-1.89) |  | 1.06 (.71-1.56) |  |  | 1.47 (1.10-1.96) |  | 1.30 (.92-1.82) |
| ***TYMS*** |  |  |  |  |  |  |  |  |  |  |  |  |  |  |
| ≤Median | 21/351 | 1.00 |  | 1.00 |  | 14/231 | 1.00 |  | 1.00 |  | 7/120 | 1.00 |  | 1.00 |
| >Median | 32/242 | 1.93 (1.09-3.43) |  | 1.22 (.61-2.44) |  | 11/109 | 1.35 (.60-3.02) |  | .51 (.19-1.43) |  | 21/133 | 2.81 (1.18-6.68) |  | 2.64 (1.00-6.95) |
| Log_2_ |  | 1.50 (1.13-1.99) |  | 1.13 (.80-1.62) |  |  | 1.47 (.95-2.27) |  | .80 (.44-1.49) |  |  | 1.58 (1.06-2.35) |  | 1.58 (.93-2.69) |
| **MRE Score**^a^ |  |  |  |  |  |  |  |  |  |  |  |  |  |  |
| –8 to ­–2 | 14/261 | 1.00 |  | 1.00 |  | 7/165 | 1.00 |  | 1.00 |  | 7/96 | 1.00 |  | 1.00 |
| –1 to 3 | 20/208 | 1.68 (.84-3.39) |  | 1.27 (.62-2.60) |  | 10/122 | 1.47 (.53-4.04) |  | 1.24 (.46-3.37) |  | 10/86 | 1.71 (.65-4.53) |  | 1.19 (.42-3.35) |
| 4 to 8 | 19/124 | 2.74 (1.30-5.78) |  | 1.85 (.62-4.04) |  | 8/53 | 3.06 (1.05-8.91) |  | 1.79 (.58-3.37) |  | 11/71 | 2.87 (1.04-7.88) |  | 2.10 (.73-6.05) |
| *Trend* |  | 1.15 (1.05-1.25) |  | 1.10 (1.01-1.21) |  |  | 1.14 (1.01-1.29) |  | 1.08 (.95-1.23) |  |  | 1.19 (1.05-1.35) |  | 1.15 (1.00-1.31) |
| *Note:* RNA counts were normalized log_2_-transformed prior to analysis. Analyses exclude women with unknown stage and stage IV breast cancer.  ^a^Multi-gene Race-associated Expression (MRE) score based on eight genes with higher scores indicating worse risk: for ACOX2 and MUC1, ≤median = 1 vs >median = ­–1; for FAM177A1, GSTT2, PSPH, PSPHL, SQLE, and TYMS, ≤median = –1 vs >median = ­1.  ^b^Adjusted for age, race (black vs. white), and codeset, as appropriate  ^c^Adjusted for age, race (black vs. white), codeset, PAM50 subtype (Luminal A, Luminal B, HER2-enriched, Basal-like, or Normal-like), and ROR-PT score (low, medium, or high), as appropriate | | | | | | | | | | | | | | |
